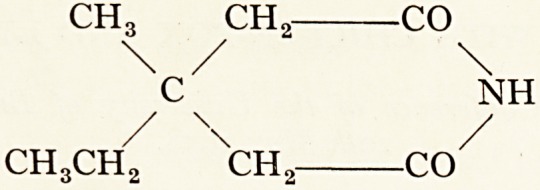# Prolonged Coma in Barbiturate Poisoning

**Published:** 1959-01

**Authors:** S. G. Flavell Matts, H. G. Handel

**Affiliations:** Department of Medicine, Frenchay Hospital, Bristol.; Department of Medicine, Frenchay Hospital, Bristol.


### PROLONGED COMA IN BARBITURATE POISONING

BY

S. G. FLAVELL MATTS, M.B. (B'ham), M.R.C.S., M.R.C.P. (Ed.)
*Senior Resident Medical Officer*

AND

H. G. HANDEL, M.B.
*Former House Physician*
*Department of Medicine, Frenchay Hospital, Bristol.*

In 1954 the recorded deaths from barbiturate poisoning in England and W[illegible] were 574 and non-fatal cases were assessed as 6,000 (Locket, 1956). In spite of [illegible] large number of reported cases in the literature, there were few in which coma [illegible] prolonged for more than a week. The *Medical Journal of Australia* (1934) mentio[illegible] a case of ten days' duration, McElligott (1955) cited another of ten days, Shulman e[illegible] (1955) described one of twelve days, Rogerson (1955) reported an eleven-day co[illegible] and Lorraine (1954) reported a case with a ten-day coma treated with massive d[illegible] of strychnine.

Greater interest has been shown in barbiturate poisoning since $\beta$-methyl-$\beta$-e[illegible] glutarimide (Megimide) was introduced into therapy by Shaw (1954) and in the [illegible] of prolonged coma reported below this drug was used with apparent benefit.

### CASE REPORT

A married woman of 46 was found unconscious in bed at 8.30 a.m. on the 31st May, 1[illegible] having taken 7 grammes of phenobarbitone the day before. There was a previous histo[illegible] depression, more marked recently and a previous attempt at suicide.

On admission she had shallow respirations, pulse 80/min., her blood pressure was 9[illegible] weak regular; there was peripheral cyanosis. She was unrousable with constricted pupils w[illegible] reacted very sluggishly to light; the fundi were normal, and there was no neck stiffness. [illegible] corneal, pharyngeal and laryngeal reflexes were absent. The tracheal reflexes were pre[illegible] the knee jerks were just elicited and the plantars responses were absent. There was some u[illegible] ation of the pharynx. There were moist sounds in all areas of the chest.

An endotracheal tube was passed immediately and Megimide 100 mgm. and Daptazol[illegible] mgm.I.V. were given at once with considerable increase in the depth of respiration. [illegible] patient remained comatose with fairly good respiratory function for another 36 hours whe[illegible] airway became obstructed with oedema of the larynx, and her respiration became impa[illegible] This necessitated emergency tracheotomy, and she was given Noradrenalin transfusion a[illegible] further 100 mgm. Megimide. This treatment produced some twitching of the muscles bu[illegible] actual convulsion. Her respiration improved and blood pressure, which had fallen to 8[illegible] rose to 100/70. A lumbar puncture was carried out, which showed the pressure to be nor[illegible] C.S.F. containing two cells and 75 mgm protein. The patient remained unconscious unti[illegible] June, 1957 when she began to move her head slightly, the peripheral reflexes became br[illegible] and the corneal reflexes returned. Her blood pressure rose to 120/80 and a slight withdr[illegible] reflex was elicited. The laryngeal reflexes returned but the pharyngeal reflex was still ab[illegible] She recovered consciousness on 8th June, 1957 when she was first able to obey simple [illegible] mands and answer simple questions.

During the time she was unconscious, her body temperature rose up to 102·6° F., and [illegible] chest became mildly infected with diminished air-entry over all areas and increase in [illegible] sounds. This was treated with terramycin ½ G. six-hourly. During this time also she [illegible] turned hourly, given intensive physiotherapy, and her trachea was *repeatedly sucked out*, [illegible] fed by a gastric tube and had Parenterovite I.V.

On discharge, tracheotomy wound had closed, no signs in chest, B.P.125/70, nysta[illegible] was fairly marked on looking to the left and right, there was mild ataxia; no defect of co-or[illegible] tion in arms or legs; there was good cerebration and she was well orientated in time and s[illegible] She was then transferred to another hospital for psychotherapy. Follow-up three months [illegible] showed her to have made a complete recovery and to have returned to normal life.

### DISCUSSION

The structural formula of Megimide is shown above and resembles that of the barbiturate ring. The mode of action was first investigated by Shaw and others (1954). They made mice and rats unconscious with pentobarbitone (60 mg/kg.) and awakened them by intraperitoneal injection of megimide (100 mg/kg.).

In rabbits, alternate intravenous doses of thiopentone and Megimide produced a prolonged alternation of sleep and waking. These observations convinced Shaw that there was a specific antagonism between it and barbiturates. Further work has supported this view (Shaw 1955, Bentel, Barlow and Ginsberg 1956, Wyke and Frayworth 1957, Turner and Hodgetts 1956, Harris 1955) but other workers have concluded that it is a functional and not a competitive barbiturate antagonist (Hahn *et al* 1956, Bottiger *et al* 1957, Frey *et al* 1956). However, all the experimental work has confirmed the drug's stimulant effect on the central nervous system and in particular on respiration. Its probable site of action has been localized to the reticular activating mechanism of the brain stem and the neighbouring structures.

Although the amount of Megimide used in this case was small (200 mgm.), it was employed at critical stages when the clinical impression was that the patient would otherwise have succumbed. Treatment of shock, of respiratory obstruction and pulmonary complications, and the constant good nursing and supervision all contributed to the patient's recovery. Phenobarbitone was the usual agent causing prolonged coma in the previously reported cases and this is probably due to its slow elimination from the body.

### SUMMARY

A case is reported of phenobarbitone poisoning with coma that lasted ten days. Megimide was found to be of value in therapy.

We would like to express our thanks to Dr. J. M. Naish for permission to publish this case.

### REFERENCES

Bentel, H., Barlow, M. B. and Ginsberg, H. (1956). *Med. Proc.*, **2**, 198.
Bottiger, L. E., Engstedt, L. and Strandberg, O. (1957). *Lancet*, **1**, 932.
Frey, H. H., Hushahn, E. W. and Soehring, K. (1956). *Arzn. Fors.* **10**, 583.
Hahn, F., Oberdorf, A. and Schunk, R. (1956). *Deut. Med. Wochens.* **81**, 1580.
Harris, T. A. B. (1955). *Lancet*, **1**, 181.
Locket, S. (1956). *Proc. Roy. Soc. Med.* **49**, 585.
Lorraine, J. M. (1954). *Bull. Soc. Med. Hosp.* Paris, **70**, 511.
McElligott, M. (1955). *Lancet*, **1**, 820.
*Med. J. of Australia.* (1934). **1**, 505.
Shaw, F. H., Simon, S. E., Cass, N., Shulman, A., Anstree, J. R. and Nelson, E. B. (1954). *Nature.* **174**, 402.
Shulman, A., Shaw, F. H., Cass, N. M., Whyte, H. M. (1955). *Brit. Med. J.* **1**, 1238.
Turner, A. W. and Hodgetts, V. E. (1956). *Aus. Vet. J.* **32**, 49.
Wyke, B. D. and Frayworth, E. (1957). *Lancet*, **2**, 1025.

## Figures and Tables

**Figure f1:**